# Modern simulation utilities for genetic analysis

**DOI:** 10.1186/s12859-021-04086-8

**Published:** 2021-05-03

**Authors:** Sarah S. Ji, Christopher A. German, Kenneth Lange, Janet S. Sinsheimer, Hua Zhou, Jin Zhou, Eric M. Sobel

**Affiliations:** 1 Department of Biostatistics, University of California, Los Angeles, 90095 USA; 2 Department of Computational Medicine, University of California, Los Angeles, 90095 USA; 3 Department of Human Genetics, University of California, Los Angeles, 90095 USA; 4 Departments of Epidemiology and Biostatistics, University of Arizona, Tucson, 85721 USA

**Keywords:** Trait simulation, Realistic genetic models, Power, Statistical genetics

## Abstract

**Background:**

Statistical geneticists employ simulation to estimate the power of proposed studies, test new analysis tools, and evaluate properties of causal models. Although there are existing trait simulators, there is ample room for modernization. For example, most phenotype simulators are limited to Gaussian traits or traits transformable to normality, while ignoring qualitative traits and realistic, non-normal trait distributions. Also, modern computer languages, such as Julia, that accommodate parallelization and cloud-based computing are now mainstream but rarely used in older applications. To meet the challenges of contemporary big studies, it is important for geneticists to adopt new computational tools.

**Results:**

We present TraitSimulation, an open-source Julia package that makes it trivial to quickly simulate phenotypes under a variety of genetic architectures. This package is integrated into our OpenMendel suite for easy downstream analyses. Julia was purpose-built for scientific programming and provides tremendous speed and memory efficiency, easy access to multi-CPU and GPU hardware, and to distributed and cloud-based parallelization. TraitSimulation is designed to encourage flexible trait simulation, including via the standard devices of applied statistics, generalized linear models (GLMs) and generalized linear mixed models (GLMMs). TraitSimulation also accommodates many study designs: unrelateds, sibships, pedigrees, or a mixture of all three. (Of course, for data with pedigrees or cryptic relationships, the simulation process must include the genetic dependencies among the individuals.) We consider an assortment of trait models and study designs to illustrate integrated simulation and analysis pipelines. Step-by-step instructions for these analyses are available in our electronic Jupyter notebooks on Github. These interactive notebooks are ideal for reproducible research.

**Conclusion:**

The TraitSimulation package has three main advantages. (1) It leverages the computational efficiency and ease of use of Julia to provide extremely fast, straightforward simulation of even the most complex genetic models, including GLMs and GLMMs. (2) It can be operated entirely within, but is not limited to, the integrated analysis pipeline of OpenMendel. And finally (3), by allowing a wider range of more realistic phenotype models, TraitSimulation brings power calculations and diagnostic tools closer to what investigators might see in real-world analyses.

## Background

Modern genome-wide association studies (GWAS) confront researchers with new computational and analytic challenges. Genetic data sets are becoming bigger and more varied. (See for example the UK Biobank [1, 2].) The size and variety of modern data sets require proper handling to ensure a quality analysis. Before mounting a study, genetic epidemiologists almost invariably evaluate the adequacy of their study design. If they propose a frequentist analysis, they seek to determine beforehand its power, type 1 error rates, accuracy and precision of estimates, coverage, and robustness to model misspecification. Given the near impossibility of deriving these results mathematically, they must resort to simulation. The whole process is time consuming and error prone due to the abundance of data and the small expected effect sizes. A single misstep can lead to false optimism or false pessimism. Since genetic studies are expensive to mount and carry important public health consequences, it is imperative that simulation studies be done realistically, accurately, and quickly.

There are a variety of existing phenotype simulators, each with its unique virtues and implementation. Three prominent examples are [3–5]. Some simulators suffer from computational limitations and bottlenecks imposed by their high-level programming language. Some simulators also require users to format, save, and then pass the simulated trait results to a separate analysis program or programs, usually written in low-level languages. Users who rely solely on these “assembly line” pipelines shoulder the growing responsibility of staying up-to-date with each program in the pipeline as it adapts to new advances in computer language, hardware, or analysis specification.

Fortunately, more recent computer languages are better suited to accommodate modern software engineering practices that protect users from common programming mistakes. Languages devised before the advent of ubiquitous parallelization and big data sets are struggling to stay relevant. On the other hand, most modern languages handle these opportunities with aplomb. Julia is a new, dynamic language that is purpose-built for high-performance computing (HPC) and widely adopted by leading computational statisticians [6, 7].

In this article we introduce TraitSimulation, an open-source Julia-based package integrated into the OpenMendel software suite [8]. OpenMendel provides a modern, comprehensive, and user-friendly pipeline for genetic analysis. TraitSimulation’s strengths are the wide range of non-normal simulation models, the flexibility to modify existing or add new simulation models, and the use of standard input and output data formats, such as PLINK. Using standard input formats, the genetic and covariate data used for trait simulation can be real or themselves simulated. Using standard output formats makes the phenotype generated by TraitSimulation compatible with a range of downstream analytical tools.

Although phenotype simulation can play a vital role in many downstream applications, including power analyses, phenotype generation itself is rarely the most time-consuming step. Usually, most time is taken by the post-simulation analyses, which may or may not be performed within our OpenMendel suite. For this reason we do not focus here on speed benchmarking of the phenotype simulation itself. Rather, we are interested in providing researchers with a tool for more flexible and perhaps more realistic phenotype simulation, while leveraging the modern language features of Julia for efficiency and ease of programming.

To demonstrate how TraitSimulation may be used as an element in the OpenMendel toolkit, we present case studies that include application of our simulation software under settings that require the wide range of possible phenotype models unique to TraitSimulation. The unified nature of the OpenMendel environment makes it easy to craft code for simulating traits conditional on real or simulated genetic and covariate data for both unrelated individuals and multi-generational pedigrees. In common with other OpenMendel packages, TraitSimulation conveniently embraces modern computing architectures and encourages reliable and efficient programming practices.

In the sections below, we first explain the advantages of using Julia to develop the OpenMendel project and demonstrate some of its key language features. We then present OpenMendel’s efficient SNP data management tool SnpArrays. Finally, we outline the trait simulation procedure with example data and results in various case studies that critically employ the simulated phenotypes in downstream analyses. The realistic trait simulation models used in these case studies are not all available in any other simulation software that we are aware of. Users are not limited to the analysis options provided in OpenMendel or Julia. After simulating the desired trait, they can call other analysis program, including popular R, C++ or Python packages, while staying within the Julia or Jupyter notebook environments. Alternatively, they can output the simulated traits to files for more customizable downstream analyses.

## Implementation

The Julia programming language provides an excellent computing environment for genetic association studies. Among Julia’s many features is its just-in-time compiler that allows the language to combine the speed and efficiencies of low-level languages such as C or C++ with the ease-of-use and understandable syntax of high-level languages such as R or Python. Julia’s speed is also enhanced by its automatic use of the tremendous parallelization built into modern CPUs. For example, Julia includes automatic instruction-level parallelism, vectorization (to carry out many mathematical operations simultaneously), and multi-threading (to have whole sections of code run in parallel); Julia even includes tools for distributed computing across massive computing clusters [9–11]. To make coding easier and more efficient, Julia also has automatic type checking (to ensure variable consistency) and multiple dispatch (in which a single function can be used with different types and amounts of data as input and still have optimal efficiency). In addition, Julia ships with a native package manager, which improves portability, ease of deployment, and reproducibility. Julia also allows users to easily maneuver between shared and distributed memory environments, including graphics processing units (GPUs). Julia’s efficiency and versatility solves the long-standing two-language problem, in which developers could quickly prototype software in higher-level languages such as R or Python but then must rewrite their prototype code in lower-level languages such as C or C++ to handle larger, real-world data sets. As genetic data evolves and grows, and more resource-intensive tools are required to perform analyses, these design features make Julia a compelling language for computational genetics.

### SNP data

Our Julia-based package SnpArrays [12] is a versatile interface to SNP data for all of our OpenMendel modules, and potentially for other packages. Users can specify SNPs of interest by name, position, minor allele frequency (MAF), or other filtering criterion provided by SnpArrays. A remarkable feature is that after reading in compressed SNP data files, SnpArrays keeps all of the genotype data compressed during its computations, such as estimation of genetic relationship matrices (GRMs) and principal components (PCs). This feature reduces RAM requirements by orders of magnitude while maintaining extremely fast performance. This is all possible because Julia allows for operations such as matrix-matrix multiplication to be defined on BitArrays, which are arrays where each element is one or two bits. This permits real analysis of biobank-scale data on commodity-level computers, which was accomplished with our software in [13].

### Trait simulation

Our new TraitSimulation program provides the broad range of underlying models listed in Table 1, including ordered multinomial models, generalized linear models (GLMs), and generalized linear mixed models (GLMM). TraitSimulation allows users to easily modify the models in Table 1 to fit their needs or to create entirely new simulation models. Table 2, whose variables are defined below, conveys the basic syntax for model construction and running the simulation under that model. This flexibility allows users to relax strict distributional assumptions imposed by many existing packages and simulate traits that do not conform to normality restrictions. Greater fidelity to trait distributions is bound to improve analysis results. Users interested in studying the robustness of their model can assess the effects of model misspecification by simulating the trait data under the hypothesized model and then analyzing the data under different models. An explicit example of the use of our software for this purpose can be seen in [13] where they found a decrease in power when analyzing ordered multinomial phenotypes under a linear or logistic regression model.

**Table 1:** Simulation models included in TraitSimulation

	Simulation model	Relatedness data	Typical application
(1)	Generalized linear models	–	Exponential-family traits
(2)	Case/control models	–	Disease status traits
(3)	Proportional hazards/odds models	–	Ordinal traits
(4)	Variance component models	GRM	Correlated normal traits
(5)	Generalized linear mixed models	GRM	Correlated non-normal traits

**Table 2:** Syntax for model construction and for the simulate function (see text for variable definitions)

Simulation model in Table 1	Model construction syntax
(1)	Model = GLMTrait($X, \beta$, dist, link)
	Model = GLMTrait($X, \beta , G, \gamma$, dist, link)
(2, 3)	Model = OrderedMultinomialTrait($X, \beta , \theta ,$ link)
(4)	Model = VCMTrait($X, \beta , vc$)
	Model = VCMTrait(formula, *df*, *vc*)
	Model = VCMTrait($X, \beta , G, \gamma , \Sigma , V$)
(5)	Model = GLMMTrait($X, \beta , vc$, dist, link)

Simulation model in Table 1	simulate Function syntax
(1, 3, 4, 5)	simulate(model)
(2)	simulate(model, Logistic = true, cutoff = 2)

To run our TraitSimulation package the typical five steps are:

1. Load the required packages: SnpArrays and TraitSimulation.
2. Read in PLINK data files via SnpArrays and estimate the GRM, if applicable.
3. Construct the simulation model, including relevant parameters such as the genetic and non-genetic predictors, the variance components, etc.
4. Call the simulation routine to sample from the constructed model.
5. Output the simulated phenotypes to a file or pass them to other analyses.

The following Julia code snippet is an example of commands used to perform the above steps for model (4) in Table 1, based on genotype data in an existing compressed file. Prospective users may interact with a comprehensive online tutorial with step-by-step instructions and sample code at [14].

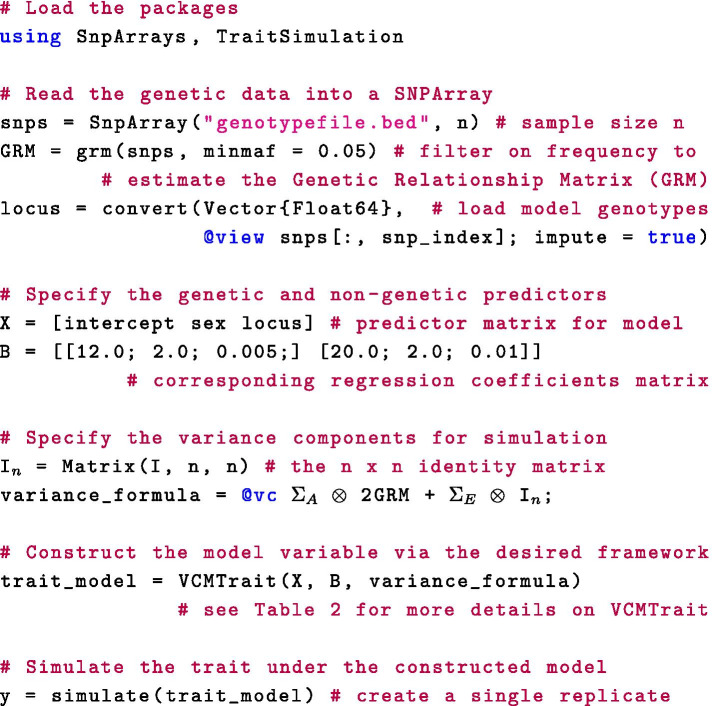



Here *X* is the matrix of predictors and *B* is the corresponding matrix of regression coefficients. The  macro provides a convenient way to specify the variance components of the model. $\Sigma _A$ is the additive genetic covariance matrix, $\Sigma _E$ is the environmental covariance matrix, $\otimes$ denotes a Kronecker product and $I_n$ is the $n \times n$ identity matrix.

Table 2 uses the same variables definitions as the above code. For each model in Table 2 the simulation procedure is also similar to the above code. Julia implements multiple dispatch that allows our simulate function to run the appropriate simulation routine even when we specify dramatically different models.

More details on running TraitSimulation under various settings, and additional step-by-step instructions for model specification, can be found in our interactive Jupyter notebooks at [14]. TraitSimulation provides users with a variety of different ways to specify the simulation model of choice. The following alternative commands to specify the genetic model may be more convenient.

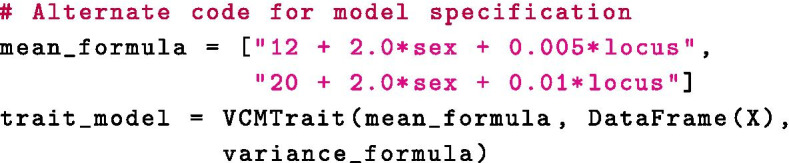



In this alternative specification, the regression coefficients in *B* are provided as a 2-element vector of formulas, one for each trait. The matrix of predictors is specified as a DataFrame with column names coordinated with those appearing in the formulas.

Another model specification mechanism provides greater flexibility for users who wish to include many SNPs without having to convert the model genotypes from the compressed SnpArray. Here, the genetic and non-genetic predictors (*G*, *X*) and the corresponding regression coefficients $(\gamma , B)$ are provided separately.

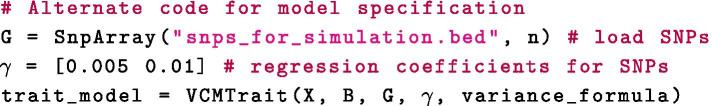



Users with many variance components may choose not to use the  macro and instead provide a list of the variance components and variance/covariance matrices:

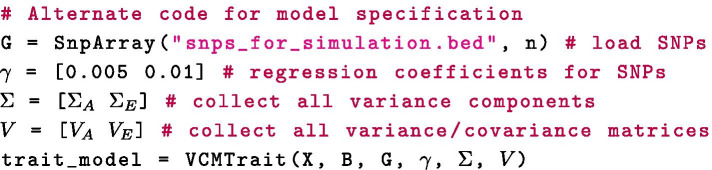



## Results

**Fig. 1:**
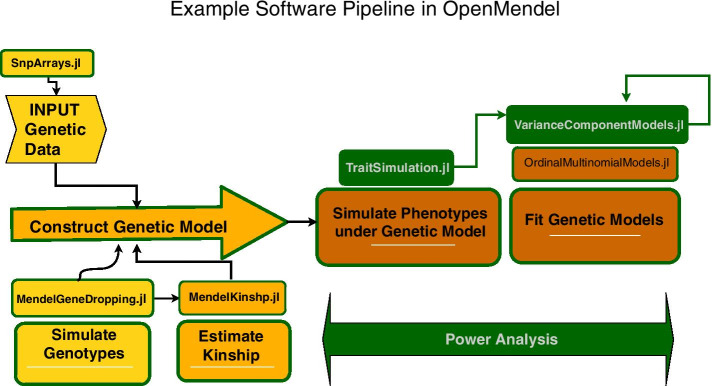
Open mendel pipeline example. TraitSimulation fits within a software pipeline to assess the power of association analysis under the variance components model of Case Study 2

In the two case studies we present below, all the generative models for trait simulation that we employ, univariate and bivariate variance components models and an ordinal multinomial model, could not currently have been built into any other trait simulation package we know. As we describe in the case studies, these are clearly the correct models for simulation given the respective data sets. Thus, TraitSimulation’s flexible model specification permits analyses that would not otherwise be available. Step-by-step, interactive Jupyter notebooks that walk the user through these case studies are available at [14]. Here, we begin by describing the statistics behind these power analysis studies and how TraitSimulation fits into the software pipeline in concert with other OpenMendel modules (see Fig. 1).

**Fig. 2:**
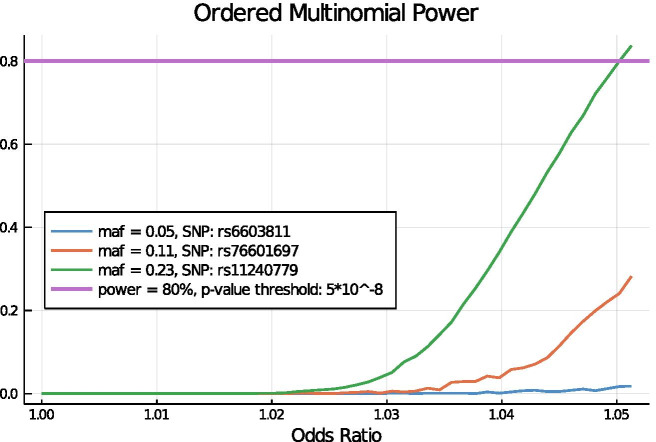
Case study 1: power under an ordinal multinomial model. This example shows the power to detect a single causal SNP in UK Biobank data with four outcome categories for disease status. Using an ordinal multinomial simulation model and the OpenMendel module for ordinal trait regression [13], we assume a single SNP as a fixed effect and control for sex and standardized age. The figure compares analysis results for three SNPs of varying MAF over 1000 simulation replicates each. For each SNP, the graph depicts the power to detect that SNP at significance level $\alpha = 5 \times 10^{-8}$. For each SNP, the effect size varies from 0 to 0.05 in increments of 0.001. On the x-axis, we exponentiate effect sizes to covert to odds ratios. See the text for a detailed description of the model

**Fig. 3:**
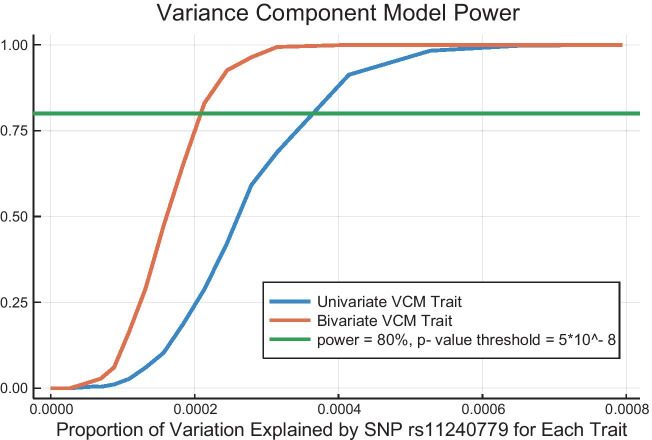
Case study 2: power under univariate and bivariate variance components models. This example shows the power to detect a single causal SNP using both univariate and bivariate variance components simulation models and the OpenMendel module for variance components analysis [8]. For each anlysis, each line in the graph depicts the power to detect a SNP with $\text {MAF} = 0.23$ using 1000 simulations at significance level $\alpha = 5 \times 10^{-8}$. The SNP effect size varies from 0 to 0.065 in increments of 0.002 in the center range (0.016–0.032) and increments of 0.005 in the two end ranges. On the x-axis, we convert the SNP MAF and effect sizes into the proportion of variation explained by the SNP. See the text for a detailed description of the model

### Statistical power

For a trait *Y* with predictor matrix $\mathbf{X}$ and genotype vector ${\mathbf {G}}_{\mathbf {s}}$, we now illustrate how to estimate the power to detect an associated SNP with effect size $\gamma$ at the pre-specified significance level $\alpha$. Specifically, we set $\alpha = 5 \times 10^{-8}$ and test the hypothesis

$$\begin{aligned} \mathbf{H} _0: \gamma = 0 \quad \text {versus}\quad \mathbf{H} _A: \gamma \ne 0 \end{aligned}$$

in our two subsequent case studies. The user also needs to specify the number of simulation replicates. In the examples presented in this article, we commence by simulating 1000 replicates of an *n*-vector of phenotypes *Y* with the specified SNP effect size $\gamma$. For each simulated trait vector, we perform a likelihood ratio test of the above hypothesis test and reject the null when the p-value falls below $\alpha$. The power for the model is estimated as the proportion of the 1000 tests rejecting the null.

#### Case study 1: power analysis for an ordinal disease

When modeling complex diseases where a binary phenotype for disease status is suboptimal, an ordered multinomial model is a powerful alternative. Our group recently demonstrated the application of an ordinal multinomial model to assess markers for association to diabetes and hypertension in the UK Biobank data [13]. Ordinal phenotypes were simulated and then fit using one of three analyses models, linear regression, logistic regression and ordered multinomial regression, to assess effects of model misspecification and show increased power under the ordered multinomial model. For the current case study, we determine the power to detect a SNP that influences an ordered categorical phenotype representing the stages of disease progression in the UK Biobank data with *n* = 185,565 subjects after data cleaning. Specifically, consider a trait *y* that takes ordered discrete values at one of *J* = 4 levels:

$$\begin{aligned} (1) \text { undiagnosed }< (2) \text { mild }< (3) \text { moderate } < (4) \text { severe }. \end{aligned}$$

Under the GLM framework, the cumulative probabilities $\alpha _{ij}$ = $\Pr (y_i \le j)$ are linked to the linear predictors via the logit link $g(\alpha _{ij}) = \eta = \log \left( \frac{\alpha _{ij}}{1 - \alpha _{ij}} \right)$. The link itself is determined by the formula

$$\begin{aligned} g(\alpha _{ij}) = \theta _j - ({\mathbf {X}}_i^T \varvec{\beta } + \gamma {\mathbf {G}}_s), \quad j = 1,\ldots , J-1, \end{aligned}$$

where the intercept parameters $\theta _1 \le \cdots \le \theta _{J-1}$ enforce the order between the categories and $\varvec{\beta }$ reflects the effects of the linear predictors under the proportional hazards model. The effect sizes can be interpreted as the expected change of the response variable on an ordered log-odds scale for each unit increase in the predictor. Figure 2 shows the resulting power curves for three SNPs with varying MAF.

#### Case study 2: power analysis for multivariate continuous traits

In this case study we carry out heritability estimation on simulated data with two variance components, one for the additive genetic variance and one for the environmental variance. TraitSimulation allows users to simulate multiple traits and more than two variance components by changing a few pertinent commands. For multivariate traits, two theoretical covariance matrices must be substituted for the additive genetic and environmental variances. Here we demonstrate how power calculations for the mixed model scale on a subset of *n* = 20,000 individuals from the same UK Biobank data used in Case Study 1. For both the univariate model ($d=1$) and multivariate ($d > 1$) mixed effect model (listed as model type (4) in Table 1), we invoke SnpArrays to estimate the kinship matrix $\widehat{{\varvec{\Phi }}}_{GRM}$ via the standard GRM formula

$$\begin{aligned} \widehat{{\varvec{\Phi }}}_{GRMij} = \frac{1}{2S} \sum _{k=1}^S \frac{(G_{ik}-2p_k)(G_{jk}-2p_k)}{2p_k(1-p_k)}. \end{aligned}$$

Here *i* and *j* are two generic individuals, *S* is the number of typed SNPs in the data, $p_k$ is the MAF of the $k{\text {th}}$ SNP, and $G_{ik} \in \{0,1,2\}$ is the number of copies of the minor allele at the $k{\text {th}}$ SNP of individual *i*. Missing genotypes are simplistically imputed on the fly as the most likely genotype given a SNP’s MAF. Finally, we make the common assumption that the residual covariance between two relatives is well approximated via the additive genetic variance times twice their kinship coefficient. The latter is taken as the corresponding entry of the GRM matrix.

Our univariate and bivariate power calculation results under the variance components model (VCM) framework appear in Fig. 3. (We used the Julia Plots package to obtain all our graphs.) In the univariate model, $\beta$ and $\gamma$ represent the non-genetic and genetic regression coefficients, respectively. We assigned 20 different values to the effect size $\gamma$ of the associated SNP or SNPs during phenotype simulation. At each $\gamma$ value, for each of 1000 replications, we tested for association using a likelihood ratio test (LRT) with significance level $\alpha = 5 \times 10^{-8}$. Symbolically, the univariate and bivariate models are

$$\begin{aligned} {\mathbf {Y}}_{n \times 1}&= {} {\mathbf {X}}\mathbf {\beta } + {\mathbf {G}}_s \gamma + {\mathbf {g}} + \mathbf {\epsilon }; \qquad\qquad \begin{array}{ll} {\mathbf {g}} \sim N({\mathbf {0}}, \sigma _A \times {\varvec{\Phi }}) \\ \mathbf {\epsilon } \sim N({\mathbf {0}}, \sigma _E \times {\mathbf {I}}_{\mathbf {n}}) \end{array}\\ vec({\mathbf {Y}}_{n \times d})&= {} vec({\mathbf {X}}{\mathbf {B}} + {\mathbf {G}}_s {\varvec{\Gamma }}) + {\mathbf {g}} + \mathbf {\epsilon }; \quad \begin{array}{ll} {\mathbf {g}} \sim N({\mathbf {0}}, \Sigma _A \otimes {\varvec{\Phi }}) \\ \mathbf {\epsilon } \sim N({\mathbf {0}}, \Sigma _E \otimes {\mathbf {I}}_n) \end{array} \end{aligned}$$

Here $\sigma _A$ and $\Sigma _A$ are the additive genetic variance and matrix, $\sigma _E$ and $\Sigma _E$ are the environmental variance and matrix, ${\varvec{\Phi }}$ is the kinship matrix, and ${\mathbf {I}}_{\mathbf {n}}$ is the $n \times n$ identity matrix. The multivariate trait model is presented in its vectorized form using the multivariate normal density, where ${\mathbf {B}}$ and ${\varvec{\Gamma }}$ are the matrix of regression coefficients for the non-genetic and genetic predictors, respectively. Kronecker products $\otimes$ are required as explained in [15].

**Table 3:** For the ordered multinomial model, power calculation runtimes in seconds

	*n* = 185,565
*k* = 1	707.8
*k* = 20	14350.2

A total of 1000 replications were performed for each combination (*k*, *n*) of the number of causal SNPs and the sample size. This was repeated for several SNP effect sizes (see Fig. 2) and the median runtimes are recorded here

**Table 4:** For the univariate and bivariate variance components model, power calculation runtimes in seconds

	*n* = 5,000	*n* = 10,000	*n* = 20,000
Univariate			
* k* = 1	72.4	202.7	815.4
* k* = 20	1422.6	4122.1	16018.4
Bivariate			
* k* = 1	215.5	354.7	978.7
* k* = 20	4207.8	7007.2	19644.8

A total of 1000 replications were performed for each combination (*k*, *n*) of the number of causal SNPs and the sample size. This was repeated for several SNP effect sizes (see Fig. 3) and the median runtimes are recorded here

### Benchmarks

Tables 3 and 4 record the median total runtimes in seconds over all 1000 replications across *k* specified SNP predictors for a sample size of *n* people, for Case Studies 1 and 2, respectively, as reported by the Julia BenchmarkTools package. All computer runs were performed on a standard 3.5 GHz Intel i9 CPU with 12 cores; they were run under Linux but we find the operating system has no appreciable effect on runtimes. As mentioned above, these power calculation runtimes are dominated by the post-simulation analyses. Thus, for variance component analyses, the runtimes scale linearly in *k*, but not in *n*, as is usual for a variance components statistical analysis. Of course, overall runtimes are linear in the number of replications chosen to perform. However, since each replication is an independent process and our programs can easily be distributed across multiple machines, using even extremely large numbers of replications, for example, for precise type 1 error estimation, is certainly feasible on a computational cluster or in the cloud.

## Conclusions

Genetic epidemiology and computational statistics are inexorably linked. The increasing size and complexity of genetic data drive improvements in algorithm design, and statistical advances push new genetic analyses. To continue this progress, we have introduced TraitSimulation, a software package that employs the Julia language to achieve impressive computational efficiencies and easy coding for a broad range of trait simulation models, including many unavailable in other simulation packages.

Simulation is a vital step in estimating the power of a proposed study to map genetic influences. To obtain the best power estimates, one must exploit all available study subjects (unrelated, sibships, parent-offspring pairs, and extended pedigrees), impute realistic genotypes (based on ethnically correct MAF, linkage disequilibrium (LD), and possibly recombination events), incorporate pertinent non-genetic predictors, and critically, simulate realistic trait values.

For example, if one is planning a family-based study and wanted to do a power analysis before collecting any data, then one would start with a collection of pedigree structures, including possibly singletons, that mimicked to the best of one’s knowledge the potential sample collection. At the founders of each pedigree one would want to simulate the genetic data using ethnic-specific allelic frequencies based on the admixture of the target population. The correct LD structure should also be maintained within these founder genomes. One way to accomplish this is to find a real genotyping or sequencing study, for example, the International Genome Sample Resource (IGSR) [16], that includes subjects in the specified ethnicities, and use the real genomes of unrelated individuals as the data for the founders of the pedigrees. Then use gene-dropping software, for example, from the OpenMendel suite, and the real human recombination map to mimic recombination events that would occur as genomes are passed from parent to child through the pedigrees. The result will be simulated but realistic genetic data for all individuals in all pedigrees, because the data reflects the appropriate allelic frequencies, LD patterns, recombination map, and relationship structure. Our TraitSimulation package can then use this data and whichever trait model you wish to study to repeatedly generate trait values. Finally, each set of simulated data would be subject to the statistical analyses that constitutes the power analysis.

The model generality, ease of use, and speed of TraitSimulation, and indeed OpenMendel as a whole, promote the agenda of modern epidemiology. TraitSimulation’s wide range of generating models improves model realism and therefore power estimation. This generality allows statistical analysis to escape the straitjacket of the Gaussian assumption by allowing case/control and ordinal disease models, and more profoundly, any GLM or GLMM structure. Our choice of the Julia computer language makes it straightforward to code software and for users to adapt existing code to fit their modeling needs. Julia enhances the speed, flexibility, and overall ease-of-use of TraitSimulation. Julia’s speed stems from its just-in-time compiler, thorough use of parallelization, and its promotion of bit-wise linear algebra operations.

TraitSimulation is part of the OpenMendel family of Julia packages [8]. OpenMendel provides an integrated suite of genetic analysis tools that rely on the standard data structure provided by SnpArrays. TraitSimulation can access other downstream analysis packages in estimating parameters and the power of new statistical tests. However, such pipeline strategies introduce extra layers of complexity and ultimately hamper analysis reproducibility. All of OpenMendel’s packages are fast, memory efficient, and user- and developer-friendly. The open-source nature of OpenMendel encourages other statisticians to extend its code base. In adding TraitSimulation to the OpenMendel family, we enable trait simulation within an integrated robust analysis pipeline. In our view, OpenMendel represents a unique and unified state-of-the-art environment for statistical genetics. We ask for your feedback and the help of the entire genetics community in perfecting OpenMendel. It or something very similar will be necessary as we face ever more massive and complex modern data sets.

## Availability and requirements

*Project name* TraitSimulation

*Project home page* https://github.com/OpenMendel/TraitSimulation.jl

*Operating systems* Linux, MacOS, Windows

*Programming language* Julia 1.0 or higher

*Other requirements* None

*License* MIT

*Any restrictions to use by non-academics* None

## Data Availability

The data used in the analysis examples in this manuscript are from the UK Biobank data repository and are publicly available, after approval by their review board, from [2]. We retrieved the data under Project IDs 48152 and 15678. Guidelines on obtaining the data used in our analyses, and step by step commands to recapitulate the analyses are available at [14].

## Abbreviations

CPU: Central processing unit
GLM: Generalized linear model
GLMM: Generalized linear mixed model
GPU: Graphics processing unit
GRM: Genetic relationship matrix
GWAS: Genome-wide association study
HPC: High performance computing
IGSR: International genome sample resource
LD: Linkage disequilibrium
LMM: Linear mixed model
LMM: Linear mixed model
LRT: Likelihood ratio test
MAF: Minor allele frequency
PC: Principal component
RAM: Random access memory
SNP: Single nucleotide polymorphism
UK: United Kingdom
VCM: Variance component model
